# Holistic needs assessment in melanoma: What does the current landscape demonstrate? A national survey

**DOI:** 10.1002/ski2.329

**Published:** 2024-02-26

**Authors:** Sami A. Raza, Diane Cannon, Faisal R. Ali

**Affiliations:** ^1^ Walsall Healthcare NHS Trust Walsall UK; ^2^ Melanoma UK Oldham UK; ^3^ St. John's Institute of Dermatology Guy's Hospital London UK; ^4^ Mid Cheshire Hospitals NHS Foundation Trust Cheshire UK

Dear Editor, Deploying and utilising a bio‐psycho‐social model of illness is the vanguard of contemporary medical practice. Coined by Engel in 1977,^1^ it promotes an interplay in health between biochemical processes, the mind, and the social determinants of health. Considering the need this, particularly in oncology, a HNA aims to achieve this model. A HNA helps to “identify and address the needs and concerns of people living with cancer to develop a personalised care and support plan”.^2^ It involves a questionnaire for patients, followed by a discussion with a clinician, to then develop a personalised care and support plan.

With over 16,000 new melanoma cases per year,^3^ we conducted a national survey to assess the use of HNAs across the United Kingdom.

We designed a semi‐structured online questionnaire which was distributed to a mailing list from a patient support group for a four‐week period between April and May 2022. 200 responses were collated from across the United Kingdom (Table 1).

**TABLE 1:** Baseline characteristics of patients who responded to the survey.

Home country of patient	England: *n* = 168 (84%)
	Scotland: *n* = 16 (8%)
	Wales: *n* = 12 (6%)
	Northern Ireland: *n* = 4 (2%)
Stage of melanoma diagnosis	Stage 0: *n* = 4 (2%)
	Stage 1: *n* = 34 (17%)
	Stage 2: *n* = 20 (10%)
	Stage 3: *n* = 64 (32%)
	Stage 4: *n* = 78 (39%)

Following the diagnosis of melanoma, the top five areas that patients would have liked advice on were emotional and psychological support (*n* = 158; 79%), physical concerns that is, pain, fatigue, side effects (*n* = 145; 72.5%), sun protection advice (*n* = 129; 64.5%), planning for future priorities that is, travelling (*n* = 127; 63.5%), with skin self‐examination and diet and nutrition both the joint fifth most popular area for advice (*n* = 122; 61% each). This generally correlated with what patients received; following the diagnosis of melanoma, the top five areas in which patients (*n* = 200) were offered practical advice on included skin self‐examination (*n* = 65; 32.5%), sun protection advice (*n* = 63; 31.5%), physical concerns that is, pain, fatigue, side effects (*n* = 60; 30%), exercise and activity pre and post‐treatment (*n* = 41; 20.5%), and patient support groups (*n* = 35; 17.5%). Interestingly, following a diagnosis, 18% (*n* = 36) were not offered any advice. Of those that were offered a HNA and who responded (*n* = 33), most (*n* = 19; 57.6%) were offered it by a clinical nurse specialist, with (*n* = 3; 9.1%) offered it by a MacMillan nurse. Interestingly, none of the patients were offered a HNA by a dermatologist nor a plastic surgeon. Our results demonstrate that 30.5% of patients (*n* = 61) did not know what a HNA is, compared to 25.5% (*n* = 51) who were unsure. This compared to 44% of patients (*n* = 88) who did know what a HNA is. Those that did know what a HNA is (*n* = 152; 76%), 72% (*n* = 110) of patients were not offered a HNA, compared to only 15.8% (*n* = 24) who were. 11.8% (*n* = 18) were unsure whether they were offered a HNA. Of 104 respondents, since being offered a HNA, with regards to it positively impacting melanoma care, 4.8% (*n* = 5) strongly agreed, 5.8% (*n* = 6) agreed, 19.2% (*n* = 20) were neutral, 5.8% (*n* = 6) disagreed, 1% (*n* = 1) strongly disagreed and 63.5% (*n* = 66) were unsure. For those that did not have a HNA (*n* = 150), the most popular reason was that 86.7% (*n* = 130) were not offered it by a clinician in the first instance. Of those that responded (*n* = 38), 63.2% (*n* = 24) stated their HNA was not regularly reviewed, compared to 10.6% (*n* = 4) whose HNAs were reviewed; 15.2% (*n* = 10) were unsure.

We believe this is the first study investigating the use of HNA in cutaneous oncology, particularly within melanoma. There is a clear need from patients that they need to solicit advice for their non‐medical needs as evidenced above, and this generally correlates to what they receive. Our study demonstrates the heterogeneity in the use of HNAs in melanoma care, with our results demonstrating that the value of HNAs is not adequately offered and/or delivered by clinicians and not fully appreciated by patients in their care. It is clear from our study that HNAs in melanoma care are not given the priority that they deserve, even though our results demonstrate that patients have non‐medical needs that must be met, as we have noted above, with the HNA providing a route for this, especially if clinicians are striving to provide a holistic model of care that caters to the bio‐psycho‐social model of health. Although there is a paucity in the literature assessing the correlation between mental health disorders in those with melanoma, a cross‐sectional study^4^ found those with metastatic melanoma had unfavourable outcomes with regards to anxiety, depression and overall quality of life.

We also note that concerted efforts are required for patient education with regards to awareness of the HNA, specifically in terms of what it entails and the benefit it can deliver to patients, with regards to meeting unmet non‐clinical needs. This would help address the gulf in awareness as to what a HNA is and what it can truly offer. Implementing a HNA, and explaining the benefits, at the outset of diagnosis and regularly reviewing this offers patients a chance to voice their concerns with regards to how their diagnosis is impacting their life and offers clinicians an opportunity to signpost patients to the correct services in order to deliver holistic care.

A notable limitation of this study is that we did not explore the disparity in patient awareness of the HNA. Only 44% of the sample knew what a HNA is, which leaves a significant disparity in those that do not know or who were unsure. Further follow up questions should have been proposed to the latter groups to ascertain the reasons for this, as presumably, they may not have understood the terminology of HNA, or its scope. Another possible reason for this is that this may be attributed to the lack of review of the HNA, with 63.2% of patients not having their HNA regularly reviewed.

We urge clinicians and the wider melanoma care team to be cognizant of non‐medical healthcare needs in patients with melanoma. Concerted efforts should be made for patient education regarding what a HNA is and the benefits it can confer. We urge decision‐makers to consider incorporating the HNA into current guidelines for the management of melanoma^5^ to provide holistic care.

## CONFLICT OF INTEREST STATEMENT

F.R. Ali is a Medical Advisor to Melanoma UK and D. Cannon was Consultant for Melanoma UK.

[Correction added on 5‐March‐2024, after first online publication, CONFLICT OF INTEREST STATEMENT was updated.]

## AUTHOR CONTRIBUTIONS

**Sami A. Raza**: Conceptualization (equal); data curation (lead); formal analysis (lead); investigation (equal); methodology (equal); writing—original draft (lead); writing—review and editing (supporting). **Diane Cannon**: Conceptualization (equal); project administration (lead); writing—review and editing (supporting). **Faisal R. Ali**: Conceptualization (lead); data curation (supporting); formal analysis (supporting); investigation (equal); methodology (equal); supervision (lead); visualization (lead); writing—review and editing (lead).

## FUNDING INFORMATION

This article received no specific grant from any funding agency in the public, commercial, or not‐for‐profit sectors

## ETHICS STATEMENT

Not applicable.

## Data Availability

Data available on request from the authors.
